# Near-infrared fluorescence laparoscopy of the cystic duct and cystic artery: first experience with two new preclinical dyes in a pig model

**DOI:** 10.1007/s00464-017-5450-z

**Published:** 2017-03-07

**Authors:** Jacqueline van den Bos, Mahdi Al-Taher, Shu Gi Hsien, Nicole D. Bouvy, Laurents P. S. Stassen

**Affiliations:** 1 Department of Surgery, Maastricht University Medical Center, Post Box 5800, 6202 AZ Maastricht, The Netherlands; 2 NUTRIM School for Nutrition, Toxicology and Metabolism, Maastricht University, Maastricht, The Netherlands

**Keywords:** Fluorescent dyes, Near-infrared fluorescence imaging, Biliary anatomy, Laparoscopic cholecystectomy, Fluorescent cholangiography

## Abstract

**Background:**

Imaging techniques that enhance visualisation of the anatomy may help prevent bile duct injury. Near-Infrared Fluorescence Imaging is such a technique. Previous experiments with ICG have shown that illumination of the extra-hepatic bile ducts is feasible. Yet, there is room for improvement in the visualisation of the target as compared to the background. Experiments with IRDye^®^ 800CW show promising results. However, this dye is too expensive for routine clinical use. The aim of this study is to test the first applicability of two newly developed preclinical dyes regarding intraoperative imaging of the cystic duct and cystic artery, compared with IRDye^®^ 800CW.

**Methods:**

Laparoscopic cholecystectomy was performed in three pigs, using a laparoscopic fluorescence imaging system. Each pig received 6 mg of one of the fluorescent dyes (1 mg/mL; IRDye^®^ 800CW, IRDye^®^ 800BK or IRDye^®^ 800NOS) by intravenous injection. Intraoperative recognition of the biliary system and cystic artery was registered at set time points. All procedures were digitally recorded, and the target to background ratio (TBR) was determined to assess the fluorescence signal.

**Results:**

With all three fluorescent dyes, the cystic artery was directly visualised. For the visualisation of the cystic duct, 15, 34 and 30 min were needed using IRDye^®^ 800BK, IRDye^®^ 800NOS and IRDye^®^ 800CW, respectively. The maximum TBR of the cystic duct was the highest with IRDye^®^ 800NOS (4.20) after 36 min, compared to 2.45 for IRDye^®^ 800BK and 2.15 for IRDye^®^ 800CW, both after 45 min. There were no adverse events.

**Conclusion:**

IRDye^®^ 800BK and IRDye^®^ 800NOS seem to be good alternatives for IRDye^®^ 800CW for the visualisation of the cystic duct and cystic artery in pigs.

Laparoscopic cholecystectomy is standard care for symptomatic gallbladder stones or acute cholecystitis, and is one of the most commonly performed surgical procedures in the Western hemisphere. The reported incidence of bile duct injuries in laparoscopic surgery is 0.4–1.4% [1, 2]. Such injuries are associated with increased morbidity, reduced survival, impaired quality of life and have economic consequences for society [2, 3]. Anatomical variations of the biliary ducts or vascular system are not uncommon and represent operative challenges and looming sources for operative complications [3, 4]. Therefore, enhanced intraoperative visualisation of the biliary anatomy is needed to reduce the incidence of bile duct injuries.

Near-infrared fluorescence (NIRF) imaging is a promising technique for real-time delineation of the perioperative anatomy. Among other applications, it is used for visualisation of the extra-hepatic bile ducts and the cystic artery in laparoscopic cholecystectomy, and visualisation of the ureter in colorectal, urological and gynaecological surgery [5–7]. In the clinical setting, ICG is the dye used for bile duct imaging, with reasonable visualisation results that makes clinical use possible. Both the extra-hepatic bile ducts and the cystic artery can be identified [8]. Subjectively however, imaging of the target is not optimal in all patients. Better contrast between target and background is expected to increase the usefulness of the technique. Adaptation in the equipment and its characteristics is one way to address this issue. Alteration in the dye or its use is another. One such alteration is the suggestion to inject the dye a day before surgery [9]. However, due to usually performed same-day admission, this is undesirable from a logistic standpoint. Applying another fluorescent dye is a further possibility.

IRDye^®^ 800CW (LICOR Biotechnology, Lincoln, United States) is an experimental dye, which in combination with laparoscopic imaging allows intraoperative visualisation of crucial anatomical structures. In a previous animal experiment, performed by our group, this dye was compared to ICG for imaging of the extra-hepatic bile ducts and the cystic artery in the pig. The main results were an earlier visualisation of the cystic duct, when using IRDye^®^ CW-800 and a higher target-to-background ratio for the cystic artery [10]. This is in line with two other studies and makes this new dye promising for future clinical use [10–12]. However, a major disadvantage of IRDye^®^ CW-800 is its cost, which is almost tenfold that of ICG. Therefore, more affordable alternatives are needed.

Recently, the manufacturer of IRDye^®^ 800CW developed two new preclinical dyes, IRDye^®^ 800BK and IRDye^®^ 800NOS, that can be produced at a cost that is comparable to commercially available ICG. In general, the three dyes have similar properties. Emission and absorption are at about the same wavelength as for ICG. The dyes differ in hydrophilic properties, with IRDye^®^ 800NOS being the least hydrophilic. This influences their excretion and their uptake in the liver and may make them more potent with regard to their imaging capabilities of the cystic duct. The aim of the present study was to evaluate the performance of these dyes as compared to IRDye^®^ 800CW for NIRF laparoscopy of the extra-hepatic bile ducts and the cystic artery in pigs using a commercially available laparoscopic fluorescence imaging system.

## Materials and methods

This study was conducted at the central animal facilities of Maastricht University (Maastricht, The Netherlands). Animals were used in compliance with the regulations of the Dutch legislation for animal research and following a protocol that was approved by the local animal ethics committee. A pig model was chosen because of its similarity with human hepatobiliary anatomy and because previously successful experiments have been conducted with NIRF imaging in pigs [10]. Three female Dutch landrace pigs, weighing 40 kg, were used for the current study. After surgery, the pigs were sacrificed by the anaesthesiologist.

### Laparoscopic fluorescence imaging system

A commercially available laparoscopic fluorescence imaging system (Karl Storz GmbH & CO. KG, Tuttlingen, Germany) was used. The NIR light source D-Light P is a xenon-based system providing illumination light for fluorescence applications in the near-infrared wavelength range. It enables the excitation of all presented dyes: IRDye 800CW (ʎEX/EM = 775/796 nm), IRDye 800BK (ʎEX/EM = 774/790 nm) and IRDye 800NOS (ʎEX/EM = 767/786 nm). A foot pedal allows the surgeon to easily switch between the two imaging modalities. All procedures were digitally recorded. For all three dyes, the same NIRF imaging settings were used.

### Characteristics and preparation of near-infrared dyes

IRDye^®^ 800CW is a tetrasulphonated heptamethine indocyanine dye. Intravenous injection is rapidly cleared by the liver and excreted into bile. It is also cleared by the kidneys and excreted into urine. The peak absorption of this dye is 775 nm and peak excitation emission at 796 nm. The molecular weight is 1090.11 Da [13, 14].

IRDye^®^ 800BK (LICOR Biotechnology, Lincoln, United States) is a newly developed dye, primarily developed for intraoperative visualisation of the ureters. This hydrophilic dye has a maximum absorption of 774 nm and a maximum emission of 790 nm. Because of its hydrophilic nature, it is primarily cleared by the kidneys. Nevertheless, being similar in structure as IRDye^®^ 800CW, some additional clearance by the liver can be expected.

IRDye^®^ 800NOS (LICOR Biotechnology, Lincoln, United States) is also a newly developed dye, less hydrophilic, mainly developed for intraoperative visualisation of the biliary system. This dye is primarily cleared by the liver and has a maximum absorption of 767 nm and a maximum emission of 786 nm.

All three dyes were prepared following instructions of the manufacturer. They were diluted in a sterile phosphate-buffered saline (PBS) solution to a concentration of 1 milligram per millilitre. Six milligrams of each dye in this dilution was prepared for intravenous injection.

### Surgical technique

Premedication consisted of intramuscular injection of azaperone 3 mg/kg, ketamine 10 mg/kg and atropine 0.05 mg/kg. Anaesthesia was induced with intravenous thiopental 10-15mg/kg. After intubation, the pigs were maintained under anaesthesia with isoflurane and oxygen. Two expert endoscopic gastrointestinal surgeons (NB and LS) performed the operations and were assisted by residents experienced in the procedure. In each pig, a laparoscopic cholecystectomy was performed, according to the Dutch Guidelines and best practice for laparoscopic cholecystectomy applying the Critical View of Safety technique [15, 16]. One dye was tested per animal using a 6 mg dose (1 mg/mL). This was twice the dose proven sufficient in an earlier study [10]. The dose was chosen to obtain maximal visualisation since our hypothesis was that a higher dose of the dye would result in better biliary imaging. Fluorescence imaging was initiated immediately after dye injection and subsequently imaging was performed intermittently in fluorescence mode and white light mode. Intra-operatively, the researcher systematically documented on a form whether the cystic duct or cystic artery could be identified, in either of the imaging modes. For agreement on the identification on the aforementioned structures, the attending surgeon was consulted. A structure was scored as ‘identified’ if its localisation was confirmed with great certainty by the experienced surgeon.

### Postoperative quantitative fluorescence analysis

The video recordings of the procedures were assessed for the degree of fluorescence illumination using OSIRIX v7.0.1 Imaging software (Pixmeo, Geneva, Switzerland). With this software, the Target-to-Background Ratio (TBR) could be determined. The TBR was defined as the mean fluorescence intensity (FI in arbitrary units, A.U.) of three points of interest in the target (cystic artery or cystic duct), minus the mean fluorescence intensity of three points of interest in the background (liver hilum), divided by the mean fluorescence intensity of three points of interest in the background. In formula: TBR = (FI of target – FI of background)/ (FI background) [8, 10]. Areas with light scattering were avoided in these points of interest.

## Results

In all experiments, the identification of the cystic duct and cystic artery with NIRF imaging was successfully conducted. Results obtained during the operation are presented in the intraoperative registration form in Table 1.

**Table 1:** Intraoperative registration form

Experiment	Injected dye (6 mg)	Visualisation of cystic artery?	Visualisation of cystic duct?	Time to identification cystic duct
1	IRDye^®^ 800BK	Yes	Yes	15 min
2	IRDye^®^ 800NOS	Yes	Yes	34 min
3	IRDye^®^ 800CW	Yes	Yes	30 min

In the first pig (IRDye^®^ 800BK), direct clear visualisation of the cystic artery was present after injecting the fluorescent dye. Already after 15 min, the cystic duct showed NIRF signal. The second pig (IRDye^®^ 800NOS) showed immediate visualisation of the cystic artery after injection of the dye. However, due to a technical problem early in the procedure, this visualisation was not recorded, and therefore no TBR could be obtained from this fluorescent cystic artery. After 34 min, the cystic duct was clearly visible. However, manipulation of the gallbladder proved to be of influence on the visualisation of the cystic duct. Too much stretching of the fragile duct prohibited influx of fluorescent dye. This was corrected at 34 min. As in the first two pigs, the cystic artery was also clearly visible in the third pig receiving IRDye^®^ 800CW dye. After 30 min, the cystic duct could be identified in the fluorescence mode.

In all three pigs, apart from the cystic duct and cystic artery, other structures became visible. The bicornate uterus was subjectively the most fluorescent in the abdomen of all three pigs. Also, the ureters, small bowel and lymph nodes were visible in fluorescence mode in all three pigs.

### Target-to-background ratio

**Fig. 1:**
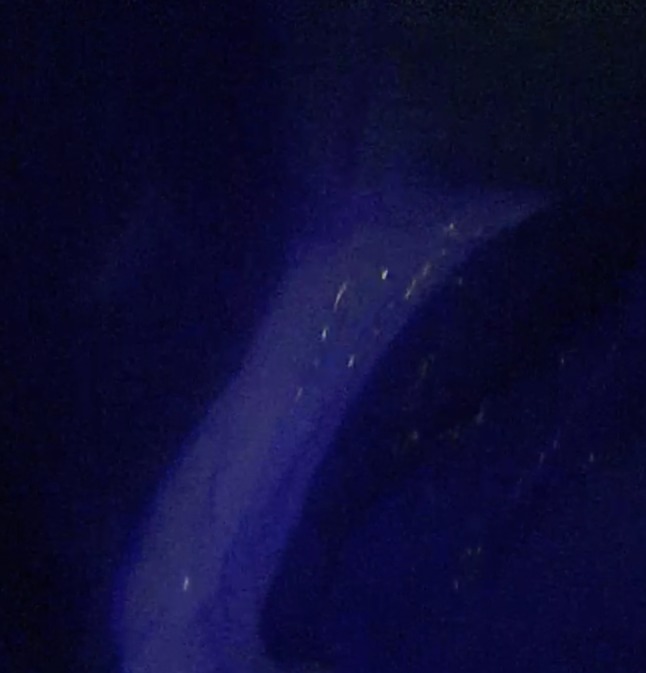
Visualisation of the cystic duct, 45 min (time point of maximal signal) after injecting 6 mg of IRDye^®^ 800BK (1 mg/mL). *Note* the little *white spots* are caused by scattering, and do not represent the fluorescent signal

**Fig. 2:**
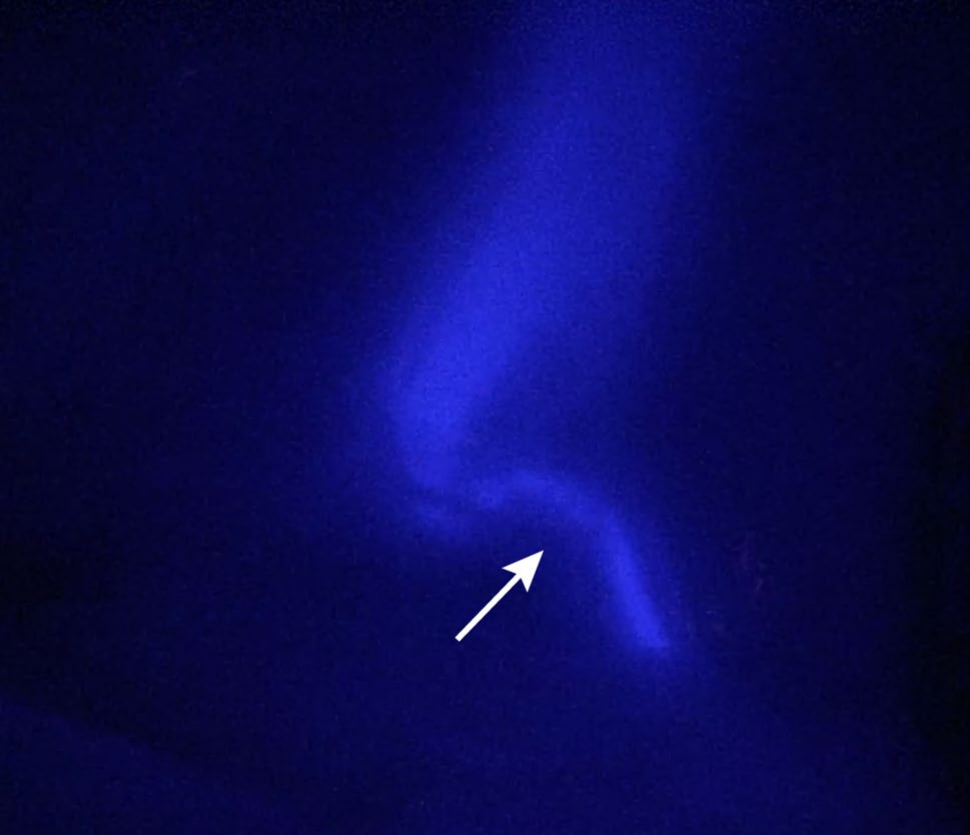
Visualisation of the cystic duct (pointed by *arrow*), and the base of the gallbladder 35 min (time point of maximal signal) after injecting 6 mg of IRDye^®^ 800NOS (1 mg/mL)

**Fig. 3:**
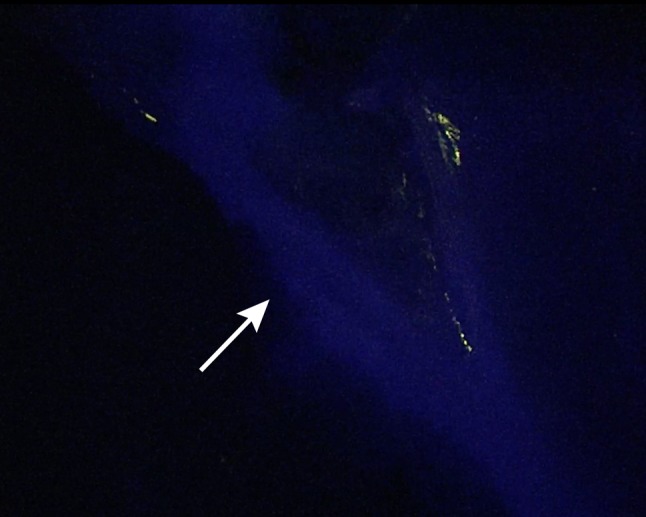
Visualisation of the cystic duct (pointed by *arrow*) and common bile duct, 45 min (time point of maximal signal) after injecting 6 mg of IRDye^®^ 800CW (1 mg/mL). *Note* the *white spots* are caused by scattering, and do not represent the fluorescent signal

Postoperatively, the video recordings were analysed by measuring the Target-to-Background Ratio (TBR). The TBR’s of cystic artery for IRDye^®^ 800BK and IRDye^®^ 800CW were 6.78 and 7.54, respectively. The TBR of the cystic artery for IRDye^®^ 800NOS could not be measured because of a technical problem regarding the video recording procedures early in the operation. The maximum measured TBRs from the three dyes for the cystic duct were 2.45 (at 45 min), 4.20 (at 35 min) and 2.15 (at 45 min) for IRDye^®^ 800BK, IRDye^®^ 800NOS and IRDye^®^ 800CW, respectively. Images 1–3 show the cystic ducts during these maximum measured TBRs. All images were made in the same fluorescence settings of the Image1 SPIES system. However, as can be seen in the images, with the IRDye^®^ 800NOS, the laparoscope was at further distance from the cystic duct. The images with the maximum TBR for each dye are shown in Fig. 1,2 and 3.

### Safety

In none of the pigs complications or adverse reactions were registered during surgery that could be attributed to one of the dyes. In particular, no alteration in heart rate and blood pressure of respiratory status was observed. Only a slightly and transient decrease in intraoperative monitoring of the Saturation of peripheral Oxygen (SpO_2_) was observed during the first minute after injection of the fluorescent dyes.

## Discussion

In the present study, the use of two new preclinical dyes for NIRF imaging of the extrahepatic bile ducts with a commercially available laparoscopic imaging system was investigated. The new dyes resemble the structure and spectral characteristics of the previously favourably tested IRDye^®^ 800CW but are much less costly, now being in the range of commercially available ICG. Both features are essential for possible future clinical application. The aim of the experiments was to perform a first assessment of the NIRF properties of the new dyes and compare these to the performance of IRDye^®^ 800CW. Eventually, these dyes could be an alternative to ICG, the only dye presently available for human use in NIRF imaging of the biliary system. This would be desirable, as with ICG, although NIRF visualisation of the bile ducts can be achieved, the observed contrast of target-to-background leaves room for improvement. Other advantages are a shorter period between injection of the dye and visualisation of the target structures and the fact that these new dyes contain no iodine, whereas some of the commercially available ICG preparations do [17]. These new dyes are therefore also applicable in patients that are known with an iodine allergy or hypersensitivity. A final benefit could be that these dyes are not only exclusively cleared by the liver, but also by the kidneys, a fact that is known from IRDye^®^ 800CW. This may make these dyes appropriate for ureter imaging.

All three dyes showed clear visualisation of the cystic artery directly after intravenous injection. Between 15 and 35 min, with all three dyes, clear visualisation of the cystic duct was achieved. Both IRDye^®^ 800BK and IRDye^®^ 800NOS caused a more prominent fluorescent signal than the earlier tested IRDye^®^ 800CW. The TBR of 2.15 found for IRDye^®^ 800CW in this study is comparable with that in an earlier study, where the TBR was 2.3 [10]. The signal from IRDye^®^ 800NOS was stronger than that from IRDye^®^ 800BK. This was expected, as IRDye^®^ 800NOS is less hydrophilic and supposed to be excreted through the bile more exclusively than IRDye^®^ 800BK. In a previous study, the TBR’s of ICG and IRDye^®^ CW800 were comparable [10]. The present results suggest that the IRDye^®^ 800NOS might be more potent for bile duct imaging. This corresponds to the subjective impression of the surgeons. Of course, these data have to be interpreted with caution and need confirmation. A possible limitation in the measurement of the TBR in this study is the not uniformly defined distance between laparoscope and cystic duct at varying time points. As shown in Images 1–3, this distance is greater in the pig with IRDye^®^ 800NOS. A larger distance between the region of interest and the laparoscope may lead to a lower fluorescence intensity [18]. However, the TBR for IRDye^®^ 800NOS was the highest of all found TBRs.

There were no adverse reactions as a result of the administration of the dye. A very early and transient decrease in oxygen saturation was measured; this is a common phenomenon with the use of intravenous fluorescent dyes [19–21]. The transient changing of the colour of the blood results in the oxygen measurements becoming falsely lower than the real value.

During the experiment, it was observed that the three dyes were all cleared by both the liver (and excreted into bile) and the kidneys (and excreted into urine). Therefore, all three dyes might be eligible for both imaging of the biliary anatomy and of the ureters, although, as previously mentioned, IRDye^®^ 800NOS is more exclusively excreted in bile and IRDye^®^ 800BK more in urine. This was confirmed by the higher TBR in the bile duct for IRDye^®^ 800NOS in this experiment. The exact behaviour of these dyes needs further study for both biliary and urinary imaging. Because of the uncertainty on the amount of the dyes that is lost in urine, the given dose was set twice as high as in a previous experiment with successful bile duct imaging with IRDye^®^ 800CW [10]. The TBR of IRDye^®^ 800CW in the present study was comparable to the previous observation which might indicate that the double dose is of no influence when using IRDye^®^ 800CW. In the present study, IRDye^®^ 800CW took longer to become visible than reported in the study by Schols et al. 2014, where the cystic duct was distinguishable after 11 min [10]. Tanaka et al. 2014 measured fluorescence in the cystic duct after around 20 min, which is comparable to the current results [11]. The different doses used may have had an influence on the time until the appearance of fluorescence. Neither can it be ruled out that another dose than the one chosen gives superior imaging. Further experiments are needed to determine optimal dosing and timing that are influenced by the pharmacokinetic properties of the dyes.

This study has two other limitations. First, because of the limited series of experiments, the results have to be interpreted with caution. Since only three pigs were used, each dye was tested only in one pig. This resulted in the fact that the failure to start video recordings early in the second pig, in which IRDye^®^ 800NOS was tested, could not be corrected in a subsequent experiment. Measurement of the visualisation of the cystic artery with TBR was therefore not possible in this pig. Another limitation is the fact that we observed influence of the manipulation with the gallbladder and cystic duct on flow of the fluorescent dye in the bile through the cystic duct in the second pig. It cannot be ruled out that earlier imaging could have been obtained with adaptation of manipulation. This may have influenced the bile flow and the detailed time measurements in the other pigs. A drawback of the procedures was the necessity to switch intermittently between NIRF- and white light imaging of the biliary system. This is inherent to the equipment used. Although likely that simultaneous white- and fluorescent light imaging is more practical, the surgeons did not experience this as hindering, and measurements could be performed at a sufficient number of time points to perform relevant observations.

This study focusses fully on the evaluation of new dyes to optimise NIRF imaging. Another way to improve NIRF imaging is adaptation of the equipment and its properties. Such approach is also very important, but outside the scope of the present experiments.

## Conclusion

The present study shows promising results on two new and affordable dyes for NIRF imaging: IRDye^®^ 800BK and IRDye^®^ 800NOS with respect to near-infrared fluorescence imaging of the cystic duct and cystic artery. At this moment, these dyes are not yet FDA approved, which also still is the case for IRDye^®^ 800CW, and are therefore not applicable for human clinical practice yet. The new dyes seem to be good alternatives for IRDye^®^ 800CW in the visualisation of the cystic duct and cystic artery in pigs. In order to be an alternative for the only dye that is at present available for human use, ICG, further studies and FDA approval are needed.
